# Clinical Characteristics and Treatment Outcomes of Patients With Advanced Germ Cell Tumor Treated at a Tertiary Cancer Center in Brazil

**DOI:** 10.1200/JGO.18.00170

**Published:** 2019-02-19

**Authors:** Vitor Florin Vasconcellos, Diogo Assed Bastos, Allan A. Lima Pereira, Gabriel Yoshiyuki Watarai, Bruno Rodriguez Pereira, Adriana de Godoy, Jamile Almeida-Silva, David Queiroz Borges Muniz, Giuliano Betoni Guglielmetti, William Carlos Nahas, Carlos Dzik

**Affiliations:** ^1^ Instituto do Cancer do Estado de São Paulo, São Paulo, Brazil; ^2^ Hospital Sírio-Libanês, São Paulo, Brazil

## Abstract

**PURPOSE:**

Reported treatment outcomes for patients with advanced germ cell tumors (aGCT) are based mainly on series from developed nations. Data from low- and middle-income countries are underrepresented.

**MATERIAL AND METHODS:**

From 2000 to 2015, a retrospective analysis identified 300 patients with aGCT treated at our institution. Kaplan-Meier methods were used for analysis of progression-free survival (PFS) and overall survival (OS) according to the International Germ Cell Consensus Classification Group (IGCCCG).

**RESULTS:**

Patients’ median age was 28 years. According to the IGCCCG, 57% had good-, 18.3% intermediate-, and 24.7% poor-risk disease. Median α-fetoprotein levels were 2.9, 243, and 3,998 ng/mL, and those of human chorionic gonadotropin were 0.4, 113, and 301.5 mUI/mL in IGCCCG good-, intermediate-, and poor-risk groups, respectively. At a median 46 months of follow-up, 93 PFS events and 45 deaths had occurred and estimated 5-year PFS and OS were 69% and 85%, respectively, including 83% and 95.3% in good-risk, 70.9% and 83.6% in intermediate-risk, and 35.1% and 62.2% in poor-risk patients, respectively. In multivariable analysis, Eastern Cooperative Oncology Group performance status ≥ 2 was a significant independent prognostic factor with a hazard ratio of 2.58 (95% CI, 1.55 to 4.29; *P* < .001) and 6.20 (95% CI, 2.97 to 12.92; *P* < .001) for PFS and OS, respectively.

**CONCLUSION:**

Brazilian patients with aGCT in this cohort had similar outcomes as patients in the IGCCCG database. In comparison with contemporary series, patients with intermediate- and poor-risk aGCT had slightly inferior PFS and OS, possibly due to a high percentage of patients with poor performance status and less use of high-dose chemotherapy.

## INTRODUCTION

Testicular cancer represents about 1% of all cancers in men and 9,310 new cases and 400 deaths due to advanced disease are expected for 2018 in the United States.^1^ In Brazil, the estimated incidence of germ cell tumor (GCT) is three to five new cases per 100,000 persons, corresponding to about 5% of all cancers in men, and about 343 deaths yearly are expected due to advanced germ cell tumor (aGCT).^2^^,^

GCT it is the most curable solid neoplasm, achieving a cure rate of 80% even in patients with advanced disease stages, defined as those with cancer metastasized to retroperitoneal lymph nodes or beyond.^3^ Unlike many other solid tumors, no new targeted therapy or immunotherapy has been added to the current state-of-the-art management of aGCT.^4^ The current therapy for advanced disease is based on the International Germ Cell Cancer Collaborative Group (IGCCCG) classification, which includes clinical, histologic, and serum tumor markers (STMs) data to stratify patients into good-, intermediate-, and poor-risk prognosis groups.^5^ Good-risk disease is treated with three cycles of bleomycin, etoposide, and cisplatin (BEP) or four cycles of etoposide and cisplatin (EP), whereas intermediate- and poor-risk cases are treated with BEP or etoposide, ifosfamide, and cisplatin (VIP) or paclitaxel, ifosfamide, and cisplatin (TIP) for four cycles.^6,7^ Patients whose disease relapses after first-line treatment can still be cured by salvage treatment with either second-line conventional-dose chemotherapy or high-dose chemotherapy (HDCT) followed by stem cell rescue.^8^

Such chemotherapy regimens are available in high- and low-income countries,^9^ which could lead one to expect no significant differences in clinical outcomes in patients with aGCT between developed and developing nations. However, other variables in addition to the treatment itself usually play a role in clinical outcomes, and no conclusion can be drawn until data from such nations are published. Unfortunately, to date, reported treatment outcomes are based mainly on series from developed nations, and data from low- and middle-income countries, such as Brazil, are underrepresented.

In this report, we characterize aGCT epidemiology and clinical pathology, evaluate validated and new prognostic factors, and also report clinical outcomes of patients with aGCT who were treated at a public tertiary cancer center in Brazil.

## MATERIAL AND METHODS

### Study Design and Eligibility Criteria

After approval by the local institutional review board, two independent authors retrospectively collected and reviewed data from electronic charts of consecutive patients with GCT treated at Instituto do Câncer do Estado de São Paulo, Brazil from 2000 to 2015.

All patients included in the analysis had histologically proven GCT and were diagnosed with advanced disease either at initial presentation or during follow-up after orchiectomy for stage I disease. Patients were deemed to have advanced disease if they met one of three criteria: (1) patients initially diagnosed with testicular GCT stage IS, II and III disease according to the seventh edition of the American Joint Committee on Cancer’s TNM staging system; (2) patients initially diagnosed with stage I testicular cancer, adequately treated with radical inguinal orchiectomy and postorchiectomy normal STMs, and whose disease later relapsed during follow-up detected by clinical, radiologic, and/or laboratorial evaluation; and (3) patients with extragonadal primary retroperitoneal and mediastinal GCT, independently of STMs or metastatic lesions.

First-line treatment of aGCT was decided on the basis of institutional protocol, multidisciplinary tumor boards, and availability of chemotherapeutic drugs in the Brazilian public health system. Our institution follow-up protocol recommendations were as follows: medical visit including history, physical examination, and STMs (namely, α-fetoprotein, β-human chorionic gonadotropin, and lactate dehydrogenase) obtained every 2 months for the first 6 months, every 3 months between 6 and 12 months, every 6 months in the second year, then annually from the third year thereafter. Abdominal and pelvis computed tomography scan associated with chest radiograph or computed tomography scan every 3 months during the first year, every 6 months during the second year, and annually during the third until at least the fifth year of follow-up. After the fifth year of follow-up, the institutional protocol suggests referring our patients to maintain routine surveillance exclusively with the primary public service.

All patients were classified according to IGCCG prognostic groups. We excluded patients who did not have histologically proven GCT and/or did not receive active treatment of advanced disease.

### Statistical Analysis

Baseline demographic, clinical, pathologic, and treatment features were summarized with descriptive statistics. For survival variables, the beginning date was defined as (1) the date of orchiectomy or the date of first biopsy with histologic confirmation of GCT (whichever occurred first) to primary testicular, retroperitoneal, or mediastinal diagnosed advanced disease; or (2) the date of first biopsy with histologic confirmation of recurred GCT or of the first day of chemotherapy (whichever occurred first) in the cases of relapsed stage I disease. Progression-free survival (PFS) was defined as time to detected disease progression or death, whichever occurred first, or end of follow-up (censored). Overall survival (OS) was defined as time to death from any cause or to end of follow-up (censored).

All data were collected between January 2017 and August 2017. Survival outcomes were analyzed using Kaplan-Meyer curves and compared by log-rank tests. Multivariate Cox regression was tested to identify prognostic factors. All statistical analyses were performed with SPSS software, version 24.0 (SPSS, Chicago, IL) and R statistical package, version 3.2.2 (https://www.r-project.org/). Significance was established as two-sided *P* < .05.

## RESULTS

### Baseline Characteristics

A total of 300 patients fulfilled the eligibility criteria. Median age at aGCT diagnosis was 28 years; all patients included were male. Most patients (n = 275; 91.7%) had a primary testicular tumor and approximately two-thirds of the patient population (n = 206; 68.3%) had nonseminoma histology (nonseminomatous GCT [NSGCT]). In our cohort, 91.7% had advanced disease at diagnosis and relapsed disease developed in 8.3% after orchiectomy. Regarding TNM staging, 130 patients (43%) had stage II, 168 patients (56%) had stage III, and only two patients (0.7%) had stage IS disease. Main baseline clinical and laboratorial characteristics are summarized in Table 1.

**TABLE 1:**
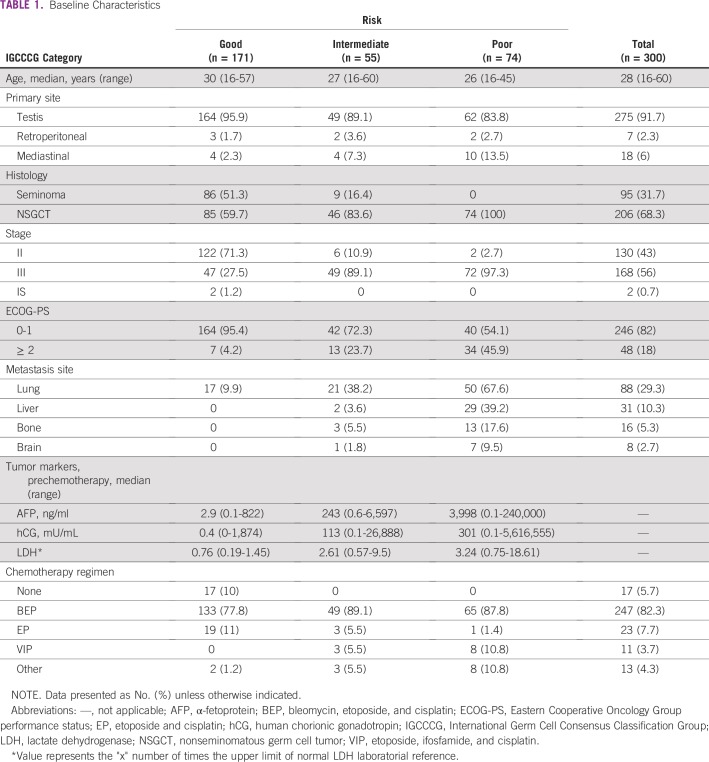
Baseline Characteristics

According to IGCCCG classification, 171 patients (57%) had good-risk disease, 55 (18.3%) had intermediate-risk disease, and 74 patients (24.7%) had poor-risk disease. In our population, almost one in five patients (n = 54; 18%) had poor performance status (PS) defined as Eastern Cooperative Oncology Group (ECOG)-PS 2, 3, or 4 before starting treatment.

### Treatment Features and Survival Outcomes

BEP for three or four cycles was the most used first-line chemotherapy regimen (n = 247; 82.3%). Other treatment protocols used in the first-line setting were four cycles of EP and VIP in 7.7% and 3.6% of patients, respectively. Seventeen patients with stage II good-risk disease did not receive chemotherapy as first-line treatment: 11 patients with NSGCT underwent nerve-sparing retroperitoneal lymph node dissection (RPLND) and six patients with pure seminoma underwent retroperitoneal radiation therapy as first-line therapy after orchiectomy.

At a median follow-up of 46 months, 93 PFS events and 45 deaths had occurred. For the entire cohort, the 5-year PFS rate was 69% and 5-year OS rate was 85%. Comparing the survival rates between different histology classifications, we found lower survival outcomes in patients with NSGCT. The 5-year PFS was 82.8% in patients with pure seminoma and 61.1% in those with NSGCT (*P* = .00043). The 5-year OS absolute difference was 21.1% (96.8% and 75.7%, respectively; *P* = .00015).

**FIG 1:**
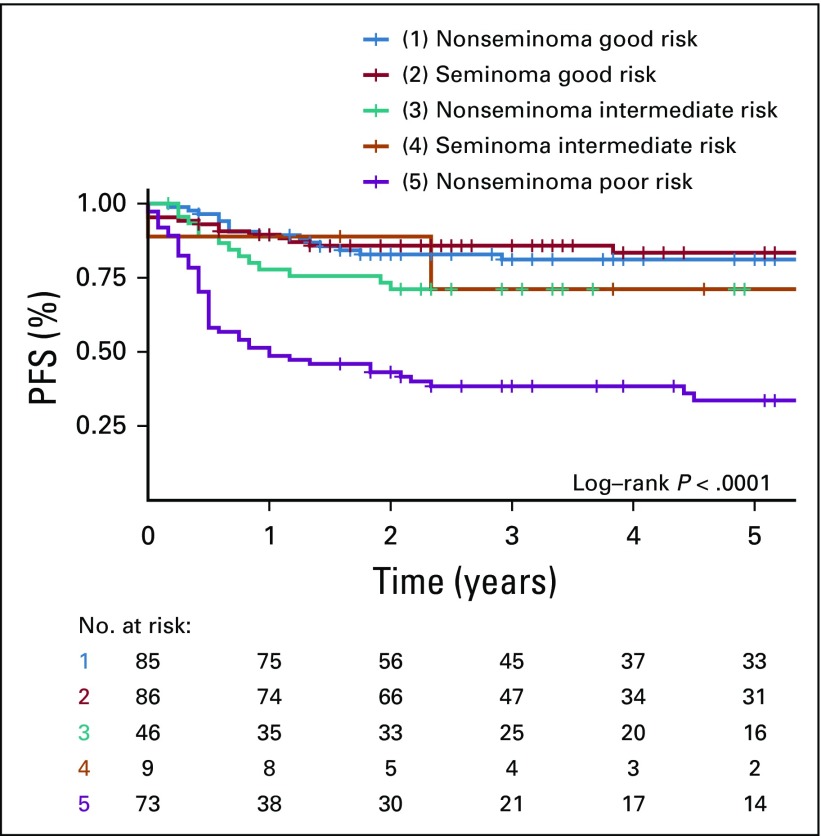
Kaplan-Meier graph of progression-free survival (PFS) and at-risk table.

**FIG 2:**
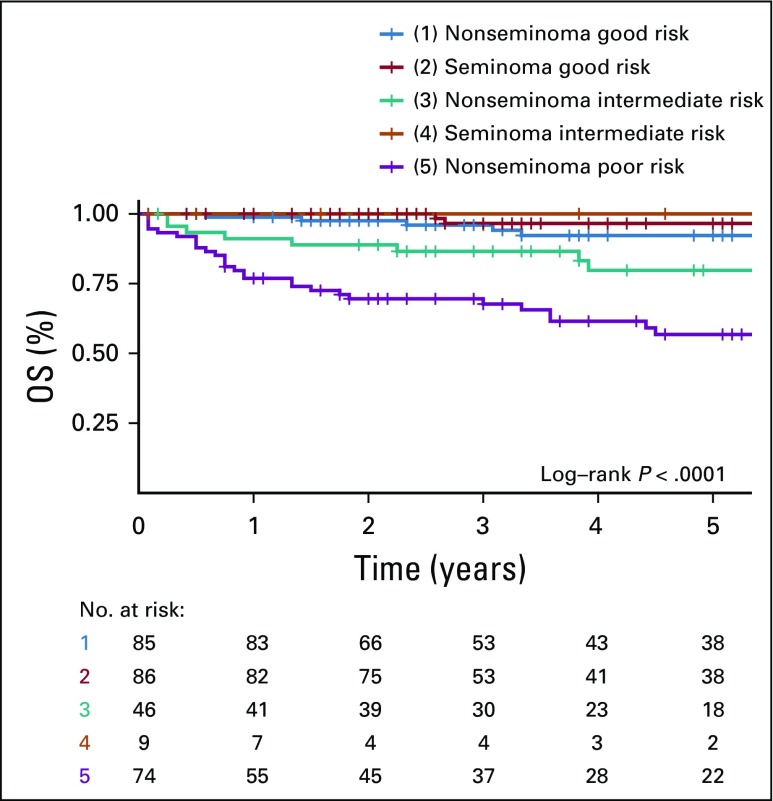
Kaplan-Meier graph of overall survival (OS) and at-risk table.

In subgroup analysis by IGCCCG prognostic groups, 5-year PFS and OS rates were 83% and 95.3% in good-risk, 70.9% and 83.6% in intermediate-risk, and 35.1% and 62.2% in poor-risk groups, respectively. Figures 1 and 2 present the survival curves by histologic and IGCCCG prognostic groups.

The pure seminoma intermediate-risk subgroup had nine patients, of whom three had complete responses and six had partial responses after first-line chemotherapy. During follow-up, three PFS events were noted and the patients experiencing these all received TIP as salvage therapy. Until the final date of analysis, one patient was receiving salvage chemotherapy, another was lost to follow-up, and a third patient had a late relapse and died 9 years after the initial diagnosis.

Ten patients (3%) died of treatment-related causes: five died of neutropenic sepsis, three of postoperative RPLND complications, one of tumoral lysis syndrome, and one of acute renal failure. Moreover, seven patients died less than 100 days from receiving aGCT diagnosis and theirs were classified as early deaths. Of note, all the latter had poor PS.

In the NSGCT subgroup, 80 patients (38.8%) had postchemotherapy RPLND as residual disease. Pathology reports indicated teratoma in 50 patients (62.5%), necrosis in 28 patients (35%), and viable GCT in only two patients (2.5%). In addition, 29 patients underwent surgical resection for other sites of suspicious lesions: nonretroperitoneal lymph nodes (n = 8; 27.5%), lung nodules (n = 3; 10%), liver metastasis (n = 1; 3.5%), and brain lesions (n = 17; 60%). Pathology reports on these 29 patients showed teratoma in 17 cases (58.5%), necrosis in eight cases (27.5%), and viable GCT in only four patients (14%). Of note, no patient with pure seminoma underwent postchemotherapy RPLND.

In terms of salvage therapy, the most frequent second-line regimen was four cycles of TIP (n = 48; 75%) and 11 patients were treated with salvage surgery. HDCT was administered in seven patients as salvage therapy, but only three patients were treated with HDCT as second-line therapy.

### Multivariate Analysis

**FIG 3:**
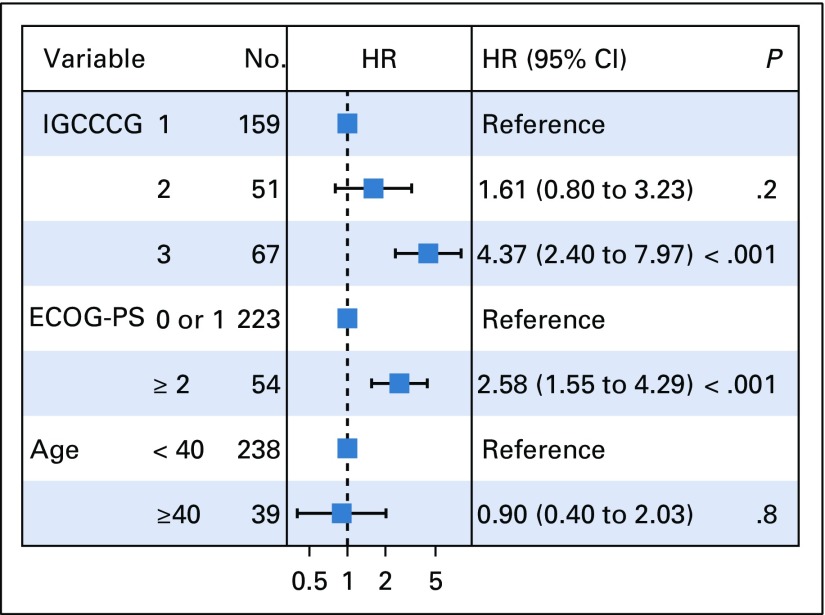
Forest plot of progression-free survival. ECOG-PS, Eastern Cooperative Oncology Group performance status; IGCCCG, International Germ Cell Consensus Classification Group. HR, hazard ratio.

**FIG 4:**
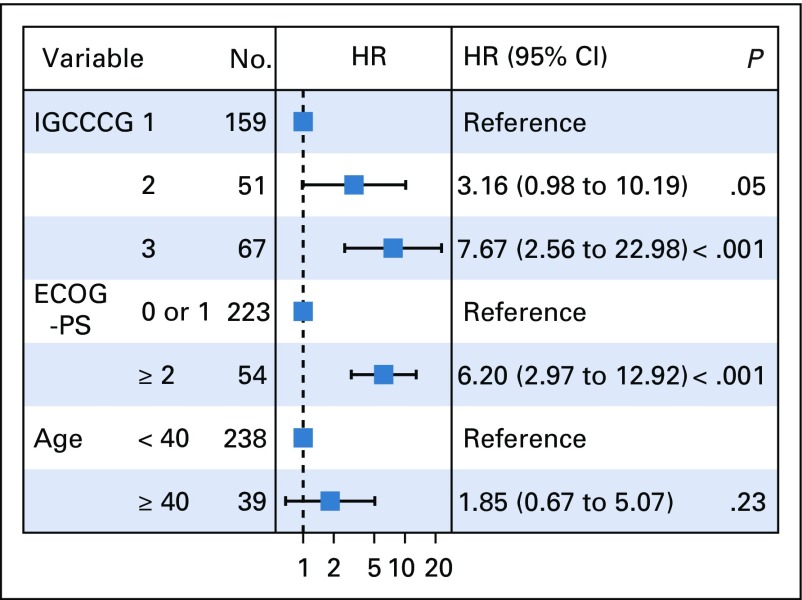
Forest plot of overall survival. ECOG-PS, Eastern Cooperative Oncology Group performance status; IGCCCG, International Germ Cell Consensus Classification Group. HR, hazard ratio.

Multivariate analysis results are shown in Figures 3 and 4. Based on the number of survival events, univariate analysis, and the medical literature, the categorical variables selected as prognostic factors were IGCCCG groups, age younger than 40 years versus ≥ 40 years, and ECOG-PS 0 or 1 versus ECOG-PS of 2 or greater.

The prognostic values of PFS and OS for patients in the IGCCCG poor-risk group were confirmed (PFS: hazard ratio [HR], 4.37 [95% CI, 2.40 to 7.97], *P* < .001; OS: HR, 7.67 [95% CI, 2.56 to 22.98], *P* < .001). In intermediate-risk patients, we observed a trend toward statistical significance for PFS (HR, 1.61; 95% CI, 0.80 to 3.23; *P* = .2) and OS (HR, 3.16; 95% CI, 0.98 to 10.19; *P* = .05). ECOG-PS of 2 or greater was a statistical significant independent prognostic factor with an HR of 2.58 (95% CI, 1.55 to 4.29; *P* < .001) and 6.20 (95% CI, 2.97 to 12.92; *P* < .001) for PFS and OS, respectively.

We also performed an exploratory analysis using age as a prognostic factor. Our cohort had 39 patients ≥ 40 years of age; therefore, the study was underpowered to adequately evaluate the prognostic impact of age for PFS (HR, 0.90; 95% CI, 0.40 to 2.03; *P* = .8) and OS (HR, 1.85; 95% CI, 0.67 to 5.07; *P* = .23).

## DISCUSSION

To our knowledge, our cohort is the largest published series of patients with aGCT treated in Latin America. Baseline clinical and demographic features are generally consistent with those reported in the literature,^6^ except that our cohort had a higher proportion of patients (18%) with aGCT with poor PS. For the entire population, 5-year PFS and OS rates were 69% and 85%, respectively, and we found that nonseminoma histology, IGCCCG classification, and ECOG-PS ≥ 2 were associated with poor survival outcomes.

Since the pivotal IGCCCG publication, prognostic groups and treatment features have been established for aGCT. The IGCCCG study^10^ consisted of a retrospective analysis of more than 3,000 patients with metastatic GCT treated with cisplatin-based chemotherapy in 10 developed countries between 1975 and 1990. The cohort 5-year OS and PFS rates were, respectively, 91% and 88% for good, 79% and 75% for intermediate, 48% and 41% for poor-risk groups.

Brazilian patients with aGCT treated in our cancer center had similar survival outcomes as patients in the IGCCCG study^10^ (Table 2). However, there are some important details that can limit the comparison between the studies. In the IGCCCG study, the heterogeneity of platinum-based regimens was higher, including some combinations that are not currently considered standard-of-care treatment.^11^ Moreover, supportive therapies used were probably less efficacious than those offered now, a higher proportion of patients were excluded due to missing information, and exploratory analysis suggested the year of treatment might have influenced the survival outcomes.^12^

**TABLE 2:**
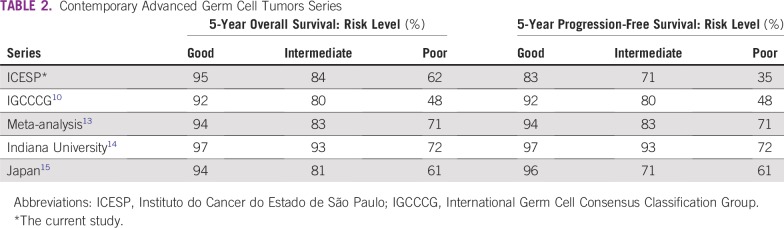
Contemporary Advanced Germ Cell Tumors Series

When compared with contemporary series, our cohort maintained similar PFS and OS values for good-risk patients, and demonstrated slightly inferior PFS and OS rates compared with intermediate- and poor-risk patients treated in developed countries (Table 2). For example, a more recent meta-analysis included a total of 1,775 patients with NSGCT treated from 1989 to 2001. Reported pooled 5-year estimated OS was 94%, 83%, and 71% for good-, intermediate-, and poor-risk patients, respectively.^13^ These increased survival rates compared with those in the IGCCCG study were more pronounced in the poor-risk group. Van Dijk et al^13^ suggested their results could be explained by the use of more effective standard treatment protocols, clinical trial selection bias, and more experience in treating patients with NSGCT.

In addition, single centers in developed countries had improved survival outcome trends over the past few decades, especially in poor-risk patients. For example, Indiana University researchers presented retrospective data from 615 patients with metastatic GCT treated at that institution from 1998 to 2012.^14^ The 5-year respective PFS and OS rates were 90% and 97% for good-risk, 84% and 93% for intermediate-risk, and 51% and 72% for poor-risk disease. The authors thought the better outcomes were possibly due to improved salvage chemotherapy.^14^

Data from less developed countries are lacking; of those that are available, most focus on clinicopathological features and per-stage survival outcomes without reporting IGCCCG prognostic classification. A Turkish, single-center, retrospective analysis included 96 patients with GCT treated between 2008 and 2013. Approximately half the cohort (n = 45; 46.9%) had stage I disease. The 5-year OS rate was 90.2% for all patients. The 2-year OS rate was 100% for patients with stage I disease, 94% for those with stage II disease, and 70.2% for those with stage III disease.^16^

Approximately half of cases (n = 12; 41%) in a small Brazilian retrospective report of 29 patients with testicular cancer from another São Paulo academic center were diagnosed with aGCT. Most (n = 9) had nonseminoma histology. All the four deaths in this cohort occurred in aGCT: three patients had NSGCT and one had pure seminoma.^17^

PS is known as a strong marker of adverse outcomes in most metastatic solid tumors.^18^ Nevertheless, data on functional evaluation are lacking in GCT, especially using validated scales such as ECOG-PS.^19^ Our study contributes to the evidenced-based importance of ECOG-PS even for a highly chemotherapy-sensitive neoplasm such as GCT. Furthermore, from a quantitative perspective, the negative prognostic impact of ECOG-PS of 2 or greater on OS had a similar magnitude as the poor-risk IGCCCG subgroup.

We believe that the high proportion of poor PS observed in our cohort reflects the high tumor burden frequently observed in patients who experience delays in diagnosis and/or referral for oncologic treatment.^20^ As with others tumors, poor PS can be attributed at least in part to socioeconomic factors, including limited knowledge about testicular cancer, long intervals between symptoms, definitive diagnostic and treatment initiation via the Brazilian public health care system.^21^

One of the greatest challenges in aGCT is state-of-the-art management of postchemotherapy residual lesions. As a national reference and academic cancer center, we had weekly, institutional multidisciplinary tumor board meetings to discuss the feasibility of complete resection, pertinent differential diagnosis, pathology reports, and the role of adjuvant chemotherapy in selected cases. Interestingly, although our cohort had a similar number of postchemotherapy RPLNDs during 15 years of analysis, we found a slightly higher proportion of teratomas and a lower prevalence of fibrosis and viable GCTs, compared with the literature.^15^

We observed a very low proportion of patients treated with high-dose chemotherapy as first salvage treatment. This is probably related to several limitations we face using HDCT at our institution, including the low socioeconomic support and educational level of patients treated in the public health system in Brazil and difficulties with the logistics for HDCT (eg, catheter placement, stem cell mobilization and collection, and referral to a hematologist).

The retrospective nature of our study reflects an inherent limitation to the results presented. Another important potential bias is that Instituto do Cancer do Estado de São Paulo is a tertiary Brazilian reference cancer center dependent exclusively on public funding and most patients included in this study were treated after 2009, when the center expanded substantially in size and the number of patients treated. On the other hand, the major strengths of our analysis are the large numbers of patients registered and treated under a uniform protocol and who represent a real-life, underreported population in a developing Latin American country.

Our study showed that in the Brazilian male population with aGCT survival outcomes were slightly inferior to reported contemporary data from developed countries, especially for patients with poor-risk NSGCT. This can be explained by the high percentage of poor PS in this cohort and less use of high-dose salvage chemotherapy. The results suggest the path for improvement efforts in GCT care and also should encourage additional developing countries studies to share challenges, solutions, and maybe collaborative efforts to achieve better outcomes for these patients.

## Data Availability

The following represents disclosure information provided by authors of this manuscript. All relationships are considered compensated. Relationships are self-held unless noted. I = Immediate Family Member, Inst = My Institution. Relationships may not relate to the subject matter of this manuscript. For more information about ASCO's conflict of interest policy, please refer to www.asco.org/rwc or ascopubs.org/jgo/site/misc/authors.html. **Honoraria:** MSD, Roche, Bristol-Myers Squibb, Janssen-Cilag, Astellas Pharma, AstraZeneca, Bayer **Consulting or Advisory Role:** Roche, Bayer, Janssen-Cilag **Research Funding:** Janssen-Cilag (Inst), Pfizer (Inst), Astellas Pharma (Inst) **Employment:** Abbott Diagnostics, Quest Diagnostics, Eisai (I) **Speakers' Bureau:** Pfizer, Janssen **Research Funding:** Pfizer (Inst) **Travel, Accommodations, Expenses:** Janssen **Consulting or Advisory Role:** Janssen-Cilag, IPSEN, Novartis **Speakers' Bureau:** Janssen Oncology **Travel, Accommodations, Expenses:** Astellas Pharma, Janssen Oncology No other potential conflicts of interest were reported.
